# Formation of the Intrathymic Dendritic Cell Pool Requires CCL21-Mediated Recruitment of CCR7^+^ Progenitors to the Thymus

**DOI:** 10.4049/jimmunol.1800348

**Published:** 2018-05-21

**Authors:** Emilie J. Cosway, Izumi Ohigashi, Karin Schauble, Sonia M. Parnell, William E. Jenkinson, Sanjiv Luther, Yousuke Takahama, Graham Anderson

**Affiliations:** * Institute for Immunology and Immunotherapy, Medical School, University of Birmingham, Birmingham B15 2TT, United Kingdom;; † Division of Experimental Immunology, Institute of Advanced Medical Sciences, University of Tokushima, Tokushima 770-8503, Japan; and; ‡ Department of Biochemistry, Centre for Immunity and Infection Lausanne, University of Lausanne, 1066 Epalinges, Switzerland

## Abstract

During αβ T cell development in the thymus, migration of newly selected CD4^+^ and CD8^+^ thymocytes into medullary areas enables tolerance mechanisms to purge the newly selected αβ TCR repertoire of autoreactive specificities. Thymic dendritic cells (DC) play key roles in this process and consist of three distinct subsets that differ in their developmental origins. Thus, plasmacytoid DC and Sirpα^+^ conventional DC type 2 are extrathymically derived and enter into the thymus via their respective expression of the chemokine receptors CCR9 and CCR2. In contrast, although Sirpα^−^ conventional DC type 1 (cDC1) are known to arise intrathymically from immature progenitors, the precise nature of such thymus-colonizing progenitors and the mechanisms controlling their thymus entry are unclear. In this article, we report a selective reduction in thymic cDC1 in mice lacking the chemokine receptor CCR7. In addition, we show that the thymus contains a CD11c^+^MHC class II^−^Sirpα^−^Flt3^+^ cDC progenitor population that expresses CCR7, and that migration of these cells to the thymus is impaired in *Ccr7^−/−^* mice. Moreover, thymic cDC1 defects in *Ccr7^−/−^* mice are mirrored in *plt/plt* mice, with further analysis of mice individually lacking the CCR7 ligands CCL21Ser (*Ccl21a^−/−^*) or CCL19 (*Ccl19^−/−^)* demonstrating an essential role for CCR7-CCL21Ser during intrathymic cDC1 development. Collectively, our data support a mechanism in which CCR7-CCL21Ser interactions guide the migration of cDC progenitors to the thymus for correct formation of the intrathymic cDC1 pool.

## Introduction

The ability of αβ T cells to recognize foreign Ags presented by self-MHC complexes takes place during T cell development in the thymus (1–3). Positive selection of immature CD4^+^CD8^+^ thymocytes triggers the expression of multiple chemokine receptors, including CCR4 and CCR7 (4, 5), enabling newly produced CD4^+^ and CD8^+^ single-positive thymocytes to migrate toward the thymus medulla (6), where T cell tolerance mechanisms take place. At this site, CD4^+^ and CD8^+^ thymocytes are screened for high-affinity αβ TCR recognition of self-antigens, including those controlled by Aire (2, 7, 8). Although high-affinity αβ TCR signaling in CD8^+^ thymocytes results in negative selection by apoptosis, CD4^+^ thymocytes can undergo two fates: either negative selection or diversion into the Foxp3^+^ T regulatory (Treg) lineage (9–11). Consequently, intrathymic elimination of self-reactive thymocytes biases conventional thymic T cell production toward self-tolerant cells, whereas Foxp3^+^ Treg development limits the autoimmune potential of developing T cells that escape thymic selection events. In the thymus medulla, specialized stromal microenvironments contain both medullary thymic epithelial cells (mTEC) and dendritic cells (DC) that express MHC class I and MHC class II (MHC II) and act as effective APCs (7, 12, 13). Importantly, αβ TCR screening for both negative selection and Foxp3^+^ Treg development can take place via direct recognition of self-antigens on mTEC themselves or following the transfer Ags to thymic DC (14–17). Thus, interplay between mTEC and DC in the thymus is important to maximize opportunities for self-antigen recognition during T cell tolerance induction.

Thymic DC are heterogeneous, consisting of both extrathymically and intrathymically derived populations (18, 19). Thus, both Sirpα^+^ conventional DC type 2 (cDC2) (20)] and plasmacytoid DC (pDC) are generated extrathymically, with their entry into the thymus providing a source of self-antigens from peripheral tissues. In contrast, Sirpα^−^ conventional DC type 1 (cDC1) arise from immature progenitors that colonize the thymus and complete their maturation intrathymically (21, 22) prior to acting as APC for mTEC-derived self-antigens (15, 23). Thus, intrathymic DC availability depends upon multiple DC subtypes. Consequently, the mechanisms that control their relative contributions to the intrathymic DC pool are important in understanding thymic tolerance. For example, thymic Sirpα^+^ cDC2 express the chemokine receptor CCR2 and are reduced in *Ccr2^−/−^* mice that display defects in negative selection (24). In addition, pDC are CCR9^+^, and *Ccr9^−/−^* mice show defects in the recruitment of pDC to the thymus and are impaired in thymocyte deletion (25). Moreover, ligands for both CCR2 (CCL2) and CCR9 (CCL25) are expressed by thymic stromal cells (26–28), highlighting the importance of thymic microenviroments in the control of thymic DC. Although these studies are important, as they explain how pDC and cDC2 are able to contribute to the intrathymic DC pool, the mechanisms that control intrathymic Sirpα^−^ cDC1 (20) are less clear. Indeed, although several studies have studied DC potential within thymic cells (21, 22, 29, 30), direct examination of the mechanisms regulating thymic cDC1 has been hindered by limitations in the identification of cDC-committed progenitors in the thymus. In contrast, stages of cDC development in peripheral lymphoid tissues are well defined (20, 31), and cDC-committed progenitors (pre-cDC) have been identified at multiple sites, including spleen and bone marrow (32–34). For example, in cell transfer experiments analyzing splenic DC development, pre-cDC with a Lin^−^CD11c^+^MHC II^−^Flt3^+^Sirpα^low^ phenotype were shown to selectively give rise to cDC progeny but not pDC or monocytes (32). Importantly, however, although such pre-cDC have been identified in peripheral tissues, their presence in the thymus has not been examined. Consequently, mechanisms regulating the entry of DC progenitors into the thymus, and the possible requirement for particular chemokine receptors in this process, have not been addressed.

In this study, we have examined development of the intrathymic DC pool in the adult mouse thymus. We find that the thymus contains a population of Lin^−^CD11c^+^MHC II^−^Flt3^+^Sirpα^low^ pre-cDC that expresses the chemokine receptor CCR7. In adult *Ccr7^−/−^* mice, we show that a selective reduction in cDC1 correlates with a reduction in thymic pre-cDC, with short-term in vivo homing assays indicating a reduced ability of *Ccr7^−/−^* pre-cDC to enter the thymus. Finally, by analyzing mice lacking expression of individual CCR7 ligands, we demonstrate a selective reduction in thymic pre-cDC and DC1 in CCL21Ser-deficient (*Ccl21a^−/−^)* but not CCL19-deficient (*Ccl19^−/−^*) mice. Collectively, our study demonstrates a mechanism in which CCR7 regulates thymic cDC1 development by controlling the intrathymic availability of pre-cDC via its ligand CCL21Ser.

## Materials and Methods

### Mice

Wild type (WT) C57BL/6 (CD45.2^+^), BoyJ (CD45.1^+^), WT C57BL/6 CD45.1^+^ CD45.2^+^, *plt/plt* (35), and *Ccr7^−/−^* (36) mice were housed at the University of Birmingham Biomedical Services Unit. All experimental procedures were approved by the Birmingham Animal Welfare and Ethical Review Body and performed in accordance with U.K. Home Office regulations. CCL19-deficient *Ccl19*^−/−^ mice (37) were housed at The University of Lausanne, Switzerland, and CCL21Ser-deficient *Ccl21a^−/−^* mice (38) were housed at The University of Tokushima, Japan. All mice were used at 8–12 wk of age.

### Abs and flow cytometry

For analysis of DC and pre-cDC, thymus and spleen samples were digested using collagenase D (Roche) and DNase I (Roche). Analysis of pre-cDC was also performed on bone marrow preparations flushed from isolated femurs and tibias. Cell suspensions were stained with Abs to the following: CD11c (N418), PDCA-1 (129C1), Sirpα (P84), CD45.1 (A20), CD45.2 (30-F11), CCR7 (4B12), MHC II (M5/114.15.2), and Flt-3 (A2F10). Analysis of DC and pre-cDC was performed after electronic gating on lineage^−^ (Lin^−^) cells using FITC-conjugated Abs to the following: CD3 (145-2C11), CD19 (eBio1D3), NK1.1 (PK136), TER119 (TER119), and B220 (RA3-6B2).

### Mixed bone marrow chimera generation

Bone marrow samples from the femurs and tibias of CD45.1^+^ WT, CD45.2^+^ WT, or CD45.2^+^ *Ccr7^−/−^* mice were T cell depleted using an anti-CD3 PE Ab and Anti-PE MicroBeads (Miltenyi Biotec). WT:WT and WT:*Ccr7^−/−^* cells were then mixed at a 50:50 ratio, and a total of 5 × 10^6^ T-depleted cells was i.v. injected into CD45.1^+^CD45.2^+^ WT host mice that had previously been lethally irradiated (two split doses of 500 rad). Mice were sacrificed after 8 wk, and tissues were harvested for flow cytometry.

### Tracking DC migration in vivo using fluorescent microbeads

Short-term tracking of DC migration in vivo was performed exactly as described (25). In brief, 200 μl of yellow/green (YG) fluorescent (505/515) carboxylate-modified microspheres (FluoSpheres, 0.2 μm diameter; Invitrogen) were i.v. injected into adult WT or *Ccr7^−/−^* mice. Forty-eight hours postinjection, thymus and spleen tissues were isolated, and bead-labeled DC subsets and pre-cDC were analyzed by flow cytometry.

### Proliferation analysis using BrdU

BrdU incorporation was used to detect proliferation of cDC. A total of 1.5 mg BrdU was injected i.p. into mice, which were sacrificed 18 h later. Thymic cell suspensions were prepared by enzymatic digestion, and cDC1 and cDC2 populations were identified as described above. To reveal BrdU incorporation, cells were permeabilized and stained using the APC BrdU Flow Kit according to the specification (BD Pharmingen).

### Statistical analysis

All analyses used GraphPad Prism 6.0. Statistical analysis was performed using unpaired Student *t* tests. Only *p* values <0.05 were identified as significant. Nonsignificant differences were not highlighted. In all figures, error bars represent SEM.

## Results

### CCR7 controls intrathymic availability of Sirpα^−^ cDC1 and their progenitors

**FIGURE 1.**
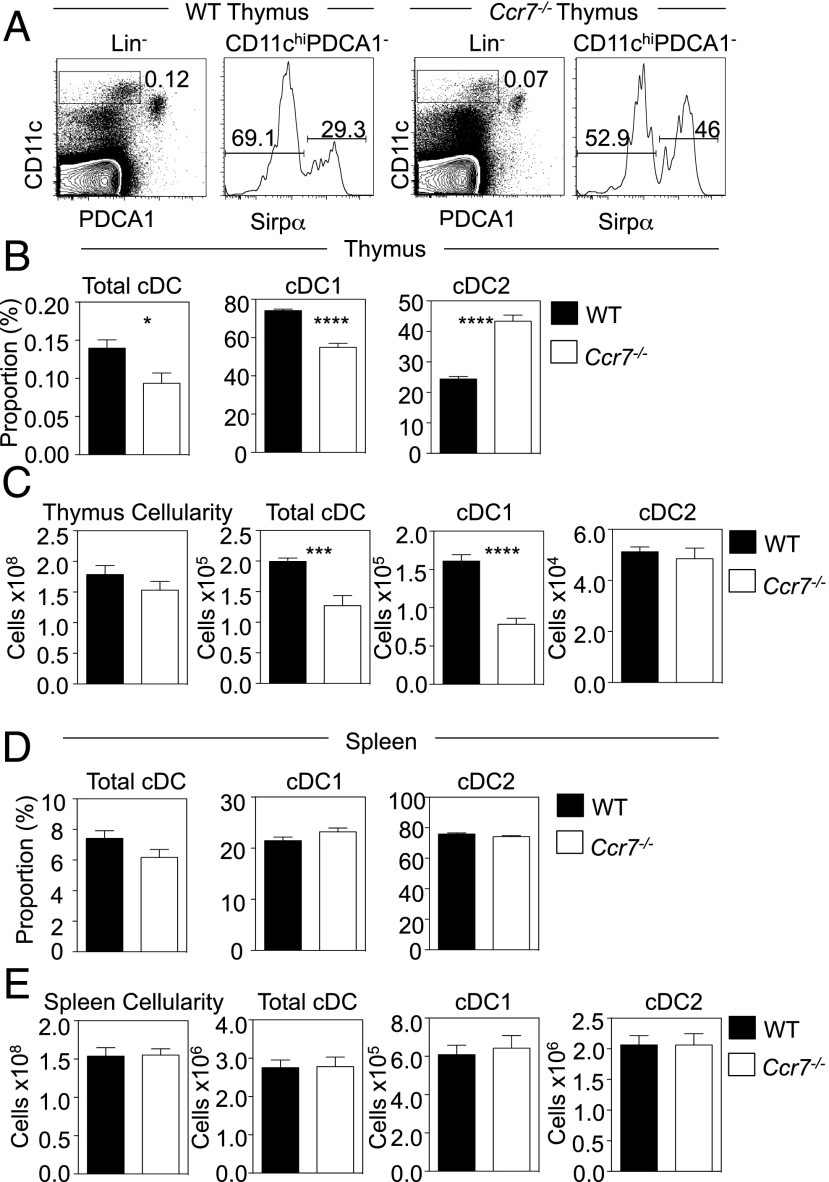
Selective reduction in intrathymic Sirpα^−^ cDC1 in *Ccr7^−/−^* mice. (**A**) Representative flow cytometric plots showing the gating strategy used to identify thymic DC. Lin^−^ refers to the exclusion of NK1.1-, CD19-, CD3-, and TER119- and B220-expressing cells. Total cDC were identified as Lin^−^CD11c^+^PDCA1^−^, which were then subdivided further to identify Sirpα^−^ cDC1 and Sirpα^+^ cDC2. Data shown are typical of at least three separate experiments. Analysis of the proportions (**B**) and absolute numbers (**C**) of DC subsets in the thymus of WT (black bars) and *Ccr7^−/−^* (open bars) mice. (**D**) and (**E**) show comparative analysis of DC proportions and numbers in the spleens of WT (black bars) and *Ccr7^−/−^* (white bars) mice. Data in (B)–(E) are from at least three separate experiments, with at least three mice per group. Error bars represent the SEM using an unpaired Student two-tailed *t* test. *p < 0.05, ***p < 0.001, ****p < 0.0001.

**FIGURE 2.**
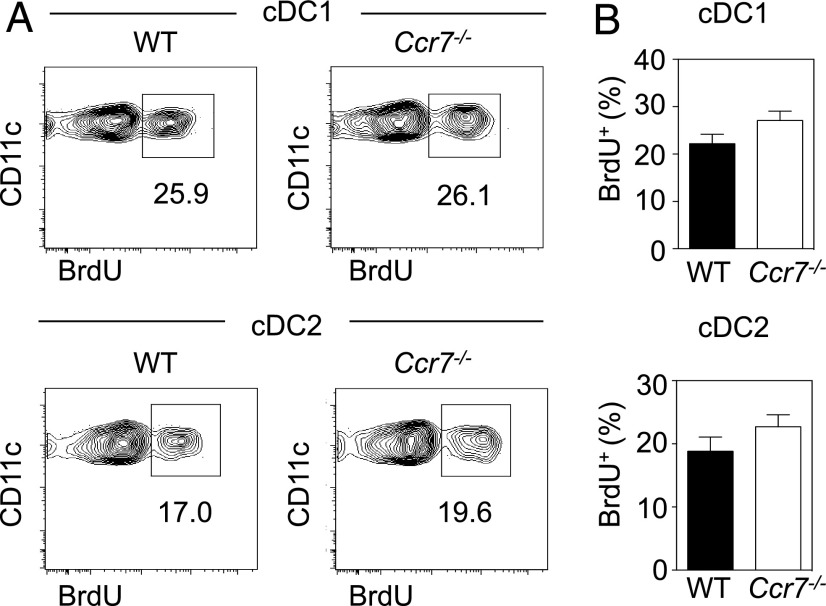
Intrathymic DC proliferation is not altered in *Ccr7^−/−^* mice. (**A**) shows analysis of BrdU incorporation in thymic cDC1 (upper panels) and thymic cDC2 (lower panels) from WT and *Ccr7^−/−^* mice. Gates for BrdU analysis were set using control mice that did not receive BrdU injections. (**B**) shows quantitative analysis of the proportions of BrdU^+^ cDC1 and cCD2 in WT (black bars) and *Ccr7^−/−^* (open bars) mice. Data are from three separate experiments, with a minimum of three mice per group.

Although chemokine receptors are known to play important roles in the recruitment of peripheral cDC2 and pDC to the thymus (24, 25, 39), mechanisms that establish intrathymic cDC1 from immature thymus-colonizing progenitors are less clear. Given that CCR7 and its ligands play an important role in the migration of DC in peripheral lymphoid tissues (40–42), we first examined the intrathymic DC pool in *Ccr7^−/−^* mice. Thymus and spleen cell suspensions from adult WT and *Ccr7^−/−^* mice were prepared, and Lineage^−^ (Lin^−^) CD11c^+^PDCA1^−^ cDC were identified by flow cytometry (Fig. 1A). Both the proportion and absolute number of intrathymic cDC were significantly reduced in *Ccr7^−/−^* mice (Fig. 1A–C). Further subdivision of total thymic cDC using Sirpα to identify Sirpα^−^ cDC1 and Sirpα^+^ cDC2 revealed that there was a significant reduction in cDC1 numbers (Fig. 1B, 1C). Importantly, splenic cDC1 proportions and numbers were comparable in WT and *Ccr7^−/−^* mice (Fig. 1D, 1E), arguing against a systemic loss of these cells in the absence of CCR7. Interestingly, cDC2 numbers were comparable in the thymus of WT and *Ccr7^−/−^* mice (Fig. 1C), indicating that the mechanisms controlling cDC2 entry to the thymus are not limited by CCR7 deficiency. In contrast, the selective cDC1 reduction in the thymus of *Ccr7^−/−^* mice suggests that CCR7 is required for the thymic entry of these cells or their progenitors. In support of this, analysis of intrathymic DC populations following in vivo BrdU administration demonstrated comparable proportions of BrdU^+^ cDC1 in both WT and *Ccr7*^−/−^ thymus (Fig. 2A, 2B), indicating that reduced thymic cDC numbers in *Ccr7^−/−^* mice are not due to reduced cell proliferation.

**FIGURE 3.**
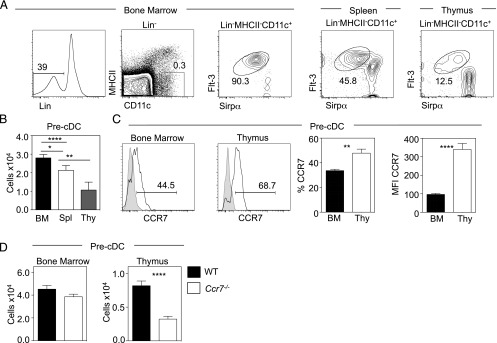
Intrathymic cDC progenitors express CCR7. (**A**) shows gating strategy used to identify Lin^−^CD11c^+^MHC II^−^Flt3^+^Sirpα^low^ pre-cDC in bone marrow, spleen, and thymus. Lin^−^ refers to the exclusion of NK1.1-, CD19-, CD3-, Ter119-, and B220-expressing cells. (**B**) Quantitation of the number of pre-cDC in indicated tissues (bone marrow [BM], spleen [Spl], and thymus [Thy]) from adult WT mice is shown. Flow cytometric plots in (**C**) show the analysis of CCR7 expression by flow cytometry on pregated Lin^−^CD11c^+^MHC II^−^Flt3^+^Sirpα^low^ pre-cDC isolated from bone marrow and thymus. Gray histograms indicate isotype control staining levels. Bar charts in (C) show proportions of CCR7^+^ pre-cDC and mean fluorescence intensity of CCR7 expression levels in pre-cDC from bone marrow (BM) (black bars) and thymus (Thy) (white bars) of WT mice. (**D**) shows numbers of pre-cDC [identified by flow cytometry as in (A)] in bone marrow and thymus preparations from adult WT (black bars) and *Ccr7^−/−^* mice (white bars). Data are from at least 10 mice of each type from three independent experiments. Error bars represent the SEM using an unpaired Student two-tailed *t* test. *p < 0.05, **p < 0.01, ****p < 0.0001. MFI, mean fluorescence intensity.

Although pre-cDC have been defined in peripheral lymphoid tissues (32–34), the precise nature of corresponding DC progenitors in thymus is still not fully clear. For example, the presence of Lin^−^CD11c^+^MHC II^−^Flt3^+^Sirpα^low^ pre-cDC (32) in the thymus has not been studied, and the relationship between these cells and other thymic DC progenitors described in additional studies is not fully clear (21, 22, 43). Interestingly, we found that Lin^−^CD11c^+^MHC II^−^Flt3^+^Sirpα^low^ pre-cDC were readily detectable in the thymus of adult WT mice (Fig. 3A), albeit at a lower frequency compared with both spleen and bone marrow (Fig. 3B). To see whether the reduction in intrathymic cDC1 in *Ccr7^−/−^* mice correlated with alterations in the frequency of pre-cDC, we first used flow cytometric analysis and anti-CCR7 Abs to examine CCR7 expression on thymic DC subsets. In agreement with earlier reports (21, 44, 45), we found that thymic cDC1 and cDC2 both expressed CCR7 (data not shown). Interestingly, pre-cDC in the thymus were also CCR7^+^, with higher CCR7 levels detectable on thymic pre-cDC, as compared with bone marrow pre-cDC (Fig. 3C). In addition, although pre-cDC numbers were comparable in the bone marrow of WT and *Ccr7^−/−^* mice (Fig. 3D), we saw a significant reduction in pre-cDC in the thymus of *Ccr7^−/−^* mice (Fig. 3D). Thus, our findings indicate that the selective loss of cDC1 in the thymus of *Ccr7^−/−^* mice is accompanied by a reduction in numbers of intrathymic CCR7^+^ pre-cDC, suggesting a role for CCR7 in the recruitment of these cells to the thymus. Furthermore, that pre-cDC are present at normal frequency in bone marrow also indicates that their reduction in the thymus is not likely due to limited availability caused by alterations in pre-cDC development at extrathymic sites.

### Thymic recruitment of pre-cDC is impaired in Ccr7^−/−^ mice

**FIGURE 4.**
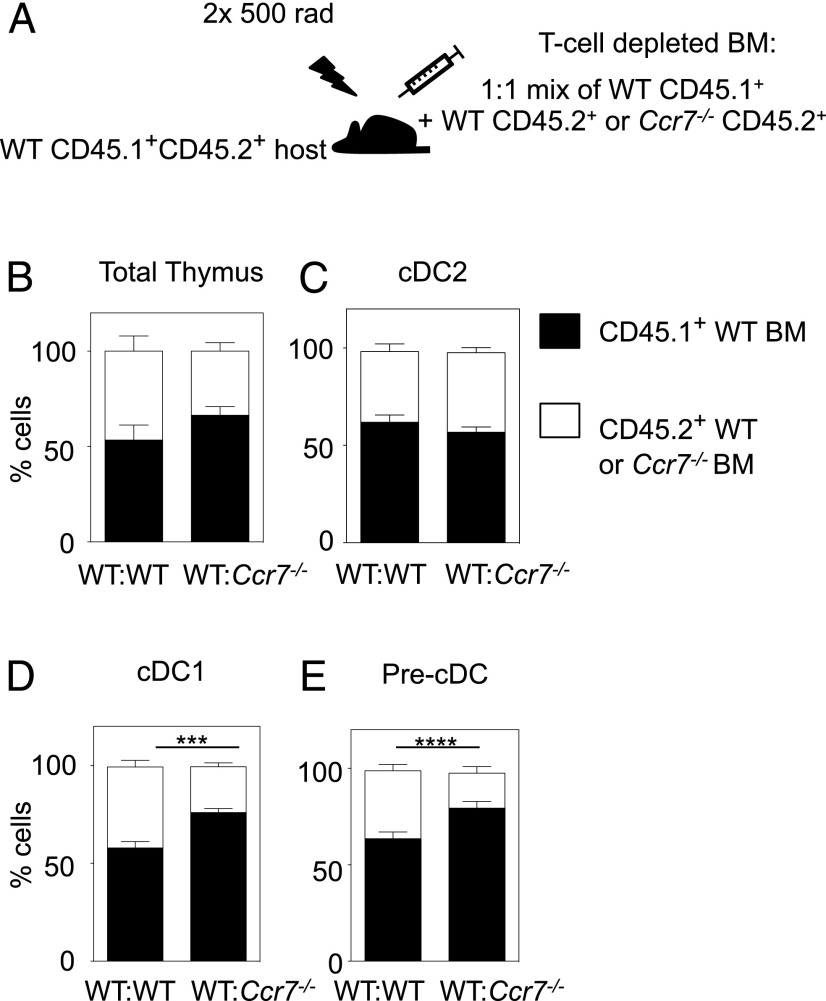
CCR7 deficiency in haemopoietic cells directly limits intrathymic cDC1 development and intrathymic pre-cDC availability. Summary of experimental approach (**A**) showing construction of mixed bone marrow chimeras using equal numbers of either WT:WT or WT:*Ccr7^−/−^* T-depleted bone marrow cells. (**B**) shows the proportion contribution of each partner to overall thymus cellularity in WT:WT and WT:*Ccr7^−/−^* chimeras as indicated, 8 wk after establishment. Similarly, and in the same chimeras, the proportion contribution of each partner in WT:WT and WT:*Ccr7^−/−^* chimeras for cDC2 (**C**), cDC1 (**D**), and pre-cDC (**E**) is shown. Data shown are representative of at least three separate experiments, each involving the generation and analysis of at least three of each chimera type. Error bars represent the SEM using an unpaired Student two-tailed *t* test. ***p < 0.001, ****p < 0.0001.

CCR7 plays an important role in the migration of newly selected CD4^+^ and CD8^+^ thymocytes into the thymus medulla (6), and the absence of CCR7 or its ligands results in disrupted medulla organization and small medullary areas (5, 46). To examine whether the defects in cDC1 and pre-cDC in *Ccr7^−/−^* mice are secondary to these alterations in medulla size, we generated bone marrow chimeras using mixtures of CD45.1^+^ WT and CD45.2^+^ *Ccr7^−/−^* progenitors, in which WT haemopoietic cells restore thymic medulla architecture (6). As controls, we established similar chimeras using mixtures of congenically marked CD45.1^+^ WT and CD45.2^+^ WT bone marrow, and all cells were transferred into CD45.1^+^CD45.2^+^ lethally irradiated hosts to allow identification of transferred WT and *Ccr7^−/−^* progeny (Fig. 4A). Mice were harvested after 8 wk, and anti-CD45.1/anti-CD45.2 Abs were used to examine chimerism within thymic cDC and pre-cDC populations. As expected, the contribution of each donor to total thymus cellularity was comparable in both WT:WT and WT:*Ccr7^−/−^* chimeras (Fig. 4B). Moreover, WT and *Ccr7^−/−^* bone marrow showed comparable contributions to intrathymic cDC2 in WT:*Ccr7^−/−^* chimeras (Fig. 4C). In contrast, we saw a significant decrease in the proportion of cDC1 generated from *Ccr7^−/−^* bone marrow in WT:*Ccr7^−/−^* chimeras (Fig. 4D). Moreover, this reduction in intrathymic cDC1 generated from *Ccr7^−/−^* marrow was accompanied by a significant reduction in the proportion of *Ccr7^−/−^*-derived pre-cDC (Fig. 4E). Thus, reductions in cDC1 and pre-cDC in unmanipulated *Ccr7^−/−^* mice still occur in the presence of WT counterparts, indicating these effects are not secondary to medulla disorganization in *Ccr7^−/−^* mice.

**FIGURE 5.**
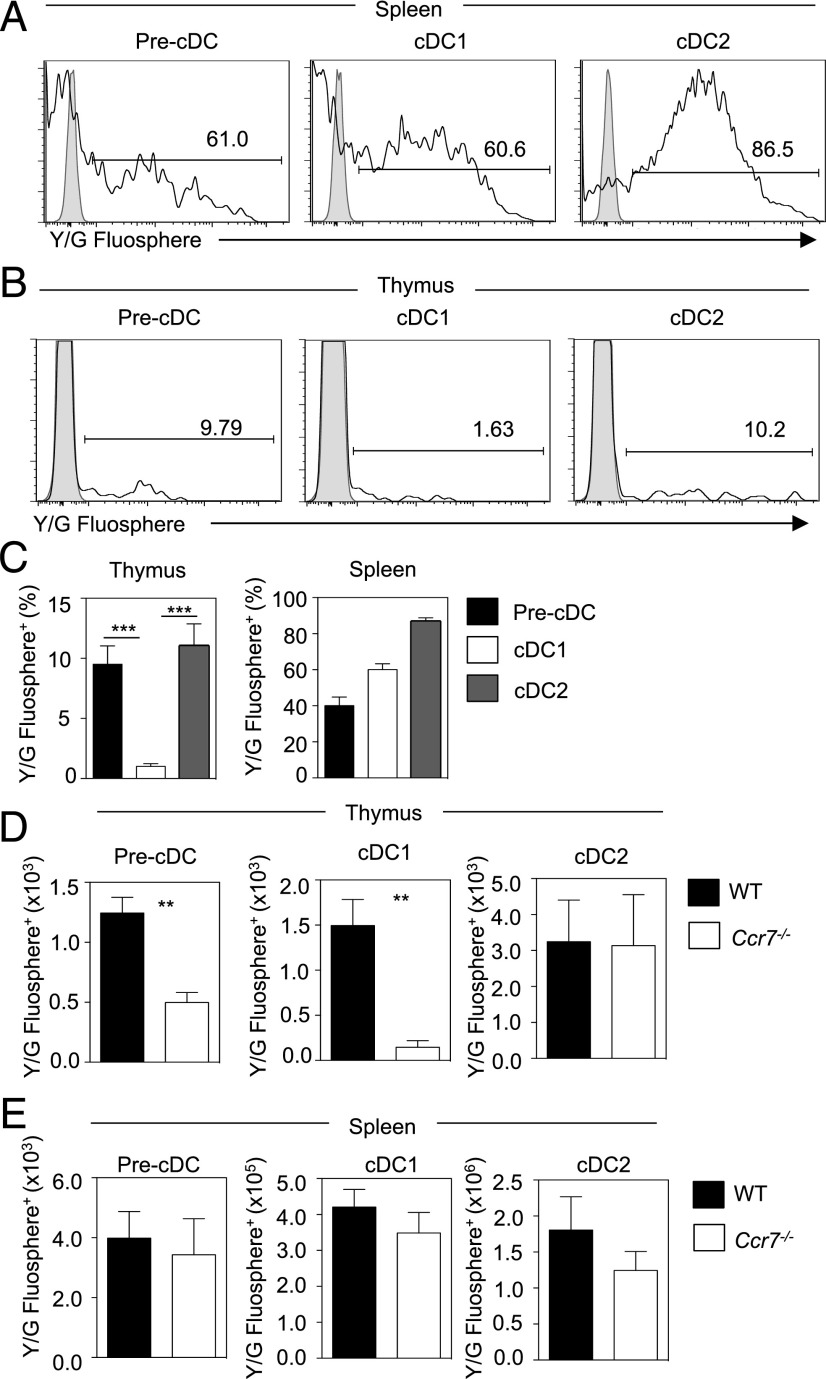
CCR7 controls the entry of cDC progenitors into thymus. To identify cells trafficking into the WT thymus, mice were i.v. injected with YG fluorescent beads (YG FluoSpheres), and tissues were harvested 48 h later. (**A**) and (**B**) show flow cytometric analysis of bead uptake in pre-cDC, cDC1, and cDC2 within the spleen (A) and thymus (B). Shaded histograms represent background fluorescence levels in PBS-injected mice. (**C**) shows proportions of bead-labeled pre-cDC, cDC1, and cDC2 in the thymus and spleen of WT mice 48 h postinjection. (**D**) and (**E**) show the number of bead-labeled DC subtypes in the thymus (D) and spleen (E) of WT (black bars) or *Ccr7^−/−^* (open bars). All data shown were obtained in at least three separate experiments, with a minimum of at least five animals of each strain. Error bars in (C) and (D) represent the SEM using an unpaired Student two-tailed *t* test. **p < 0.01, ***p < 0.001.

To directly examine the recruitment of pre-cDC to the thymus in the steady-state, we adopted a short-term homing assay used previously to examine pDC entry to the thymus, in which migratory DC are labeled by uptake of fluorescent microbeads (25). Thus, WT mice were i.v. injected with YG-labeled microbeads, and splenic and thymic DC populations were analyzed for YG labeling 2 d postinjection. As expected following i.v. transfer into WT mice, YG^+^ cells were clearly detectable within all cDC1, cDC2, and pre-cDC populations in the spleen (Fig. 5A, 5C). Interestingly, we saw differential labeling of DC populations in the thymus. Thus, ∼10% of intrathymic cDC2 were YG^+^ (Fig. 5B, 5C), consistent with the extrathymic origin of these cells. In contrast, very few (1–2%) of intrathymic cDC1 were labeled YG^+^ following i.v. microbead injection. This low frequency of bead uptake by intrathymic cDC1 is in line with their intrathymic generation and is also indicative that this labeling approach does not readily label thymic DC in situ, perhaps because of the blood–thymus barrier (47). Importantly, analysis of intrathymic pre-cDC in the same mice showed that ∼10% of these cells were YG^+^, indicating their migration to the thymus from peripheral sites (Fig. 5A, 5C). Next, when we compared YG-labeled DC populations in tissues from WT and *Ccr7^−/−^* mice after i.v. microbead transfer, we saw a significant reduction in the numbers of both YG^+^ pre-cDC and cDC1 in the thymus of *Ccr7^−/−^* mice (Fig. 5D). This was not due to differential cell labeling between strains, as no differences in the numbers of YG^+^ pre-cDC and cDC1 were seen in the spleens of WT and *Ccr7^−/−^* mice (Fig. 5E). Moreover, and consistent with unaltered cDC2 numbers in *Ccr7^−/−^* mice (Fig. 1), numbers of YG^+^ cDC2 in the thymus of WT and *Ccr7^−/−^* mice were comparable (Fig. 5D). Thus, by tracking the steady-state migration of DC subsets using short-term in vivo homing assays, our findings indicate that Lin^−^CD11c^+^MHC II^−^Flt3^+^Sirpα^low^ pre-cDC enter the thymus from the periphery and that this process is reduced in the absence of CCR7.

### CCL21, but not CCL19, controls intrathymic DC pool formation

CCL19, CCL21Ser, and CCL21Leu represent the three known functional chemokine ligands for CCR7 (48). As the genes encoding both CCL19 (*Ccl19*) and CCL21Ser (*Ccl21a*) are expressed in multiple thymic stromal cell types (49–51), we next investigated whether the requirement for CCR7 in intrathymic cDC1 development mapped to specific chemokine ligand requirements. Initially, we examined intrathymic DC in *plt/plt* mice, in which expression of both *Ccl19* and *Ccl21a* is absent (35). Thus, freshly isolated thymus tissue from adult WT and *plt/plt* mice was enzymatically digested, and intrathymic Sirpα^−^ cDC1 and Sirpα^+^ cDC2 DC subsets were identified by flow cytometry.

**FIGURE 6.**
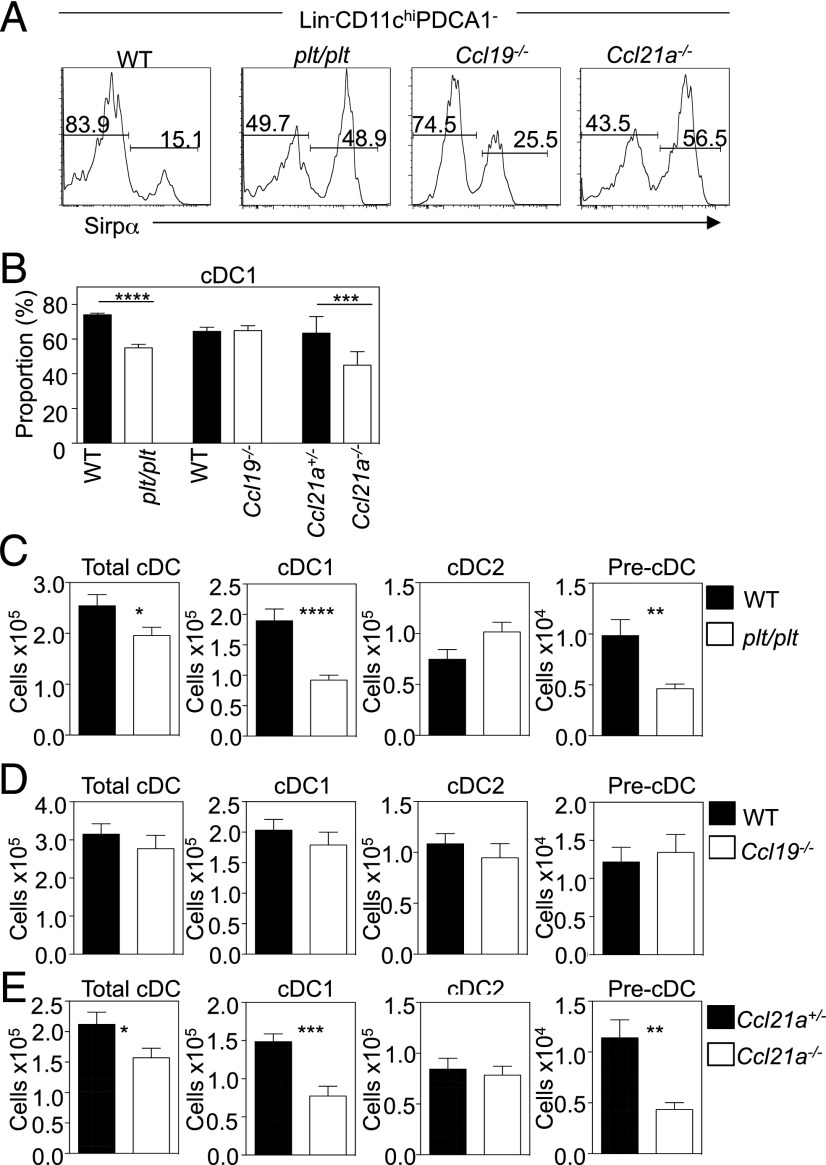
A CCR7-CCL21Ser axis regulates intrathymic cDC1 and their progenitors. (**A**) is a representative example of flow cytometric analysis of adult thymus preparations from the indicated mouse strains to show Sirpα expression in pregated Lin^−^CD11c^hi^PDCA1^−^ total cDC. (**B**) Quantitative analysis of the proportions of Sirpα^−^ cDC1 in *plt/plt*, *Ccl19^−/−^*, and *Ccl21a^−/−^* mice (black bars), compared with control mice (open bars). (**C**)–(**E**) show numbers of total cDC, Sirpα^−^ cDC1, Sirpα^+^ cDC2, and Lin^−^CD11c^+^MHC II^−^Flt3^+^Sirpα^low^ pre-cDC in adult thymus preparations from *plt/plt* [(C), white bars], *Ccl19^−/−^* [(D), open bars], and *Ccl21a^−/−^* mice [(E), open bars], compared with controls (black bars). All analysis was obtained from a minimum of nine mice per strain across at least three independent experiments. Error bars represent the SEM using an unpaired Student two-tailed *t* test. *p < 0.05, **p < 0.01, ***p < 0.001, ****p < 0.0001.

Consistent with the requirement for CCR7 and the phenotype of *Ccr7^−/−^* mice, *plt/plt* mice showed a reduction in the absolute numbers of total thymic cDC and a selective reduction in the proportion and numbers of cDC1 (Fig. 6A–C). In addition, we also saw a significant reduction in the absolute numbers of pre-cDC in *plt/plt* mice (Fig. 6C). Thus, for intrathymic DC populations, *plt/plt* mice essentially mirror the effects seen in *Ccr7^−/−^* mice. Next, to examine the requirement for individual CCR7 ligands in thymic DC development, we examined *Ccl19^−/−^* and *Ccl21a^−/−^* mice that individually lack expression of the CCR7 ligands CCL19 or CCL21Ser. Interestingly, no alterations in the numbers and proportions of cDC1, cDC2, and pre-cDC were seen in the thymus of *Ccl19^−/−^* mice (Fig. 6A, 6B, 6D). In contrast, analysis of *Ccl21a^−/−^* mice showed alterations in thymic DC populations. In particular, we saw a reduction in the number of total cDC (Fig. 6E) that was caused by a specific reduction in both the proportion and number of cDC1 (Fig. 6A, 6B, 6E). Moreover, pre-DC were also reduced in the thymuses of *Ccl21a^−/−^* mice (Fig. 6E). Thus, analysis of mice that lack CCR7 ligands either individually or in combination indicates that although CCL19 is dispensable, CCL21Ser plays an essential role in controlling the intrathymic development of cDC1.

## Discussion

In the thymus medulla, interactions between mTEC, DC, and newly selected thymocytes are essential for both negative selection and Foxp3^+^ Treg generation, which represent key aspects of T cell tolerance. Although thymic DC are known to be heterogeneous, the mechanisms that control formation of the intrathymic DC pool from its constituent components of peripherally derived pDC and cDC2 and intrathymically produced cDC1 are unclear. Given that chemokine receptors play important roles in the thymic recruitment of pDC and cDC2 (24, 25), we investigated their potential role in the development of cDC1 in the thymus. In particular, given the role of CCR7 in both thymocyte migration and DC migration in peripheral lymphoid tissues, we examined the role of this chemokine receptor and its ligands during development of the intrathymic DC pool.

In this article, we show that the thymus of *Ccr7^−/−^* mice has a selective defect in the frequency of cDC1. Mixed bone marrow chimeras show this defect maps to CCR7 expression by haemopoietic cells and is not an indirect consequence of the medullary disorganization seen in these mice. Furthermore, we show that a pre-cDC subset, previously described only in peripheral lymphoid tissues (32), is present in the thymus and expresses CCR7. Moreover, such pre-cDC are reduced in the thymus of *Ccr7^−/−^* mice, with in vivo migration assays indicating this deficiency is caused by their reduced capacity to enter the thymus. Thus, the contribution of cDC1 to the intrathymic DC pool occurs via a mechanism involving CCR7-mediated recruitment of pre-cDC. This requirement for CCR7 by cDC1 draws parallels with the respective requirements of pDC for CCR9 and cDC2 for CCR2 (24, 25) and extends our understanding of the importance of chemokine receptors in thymic DC development. In addition, our finding that thymic cDC1 are altered in *CCl21a^−/−^* but not *Ccl19^−/−^* mice highlights the importance of individual chemokines for thymic DC, with the CCR7 ligand CCL21Ser playing an essential role in intrathymic cDC1 development. Recently, another study reported that increased DC apoptosis in *Ccr7^−/−^* mice resulted in alterations in their intrathymic DC (44). However, although this study did not examine DC progenitors and mechanisms of their thymus entry, it is important to note that the cDC1 deficiency we describe in this article is accompanied by a reduction in intrathymic CCR7^+^ pre-cDC. Also, during intrathymic DC development, DC progenitors may downregulate CCR7 as they progress to an immature MHC II^low^ DC stage, which is followed by phases of steady-state maturation in the thymus that involve MHC II upregulation and the re-expression of CCR7 (45). Taken together, these observations suggest that CCR7 may play multiple roles at different stages during thymic DC development, including colonization by migrant DC progenitors, and subsequent intrathymic survival and/or maturation of their cDC1 progeny. This scenario is perhaps similar to the multiple roles played by CCR7 during conventional αβ T cell development in the adult thymus that include lymphoid progenitor colonization and cortex-to-medulla migration of positively selected thymocytes (52–55). In addition, that the reduction in pre-cDC in *Ccr7^−/−^* mice does not result in increased compensatory proliferation in either these cells or their cDC1 progeny (data not shown) may also indicate a limited availability of intrathymic growth factors for DC and/or their progenitors.

Our findings are also significant in relation to the intrathymic developmental potential of pre-cDC defined by a Lin^−^CD11c^+^MHC II^−^Flt3^+^Sirpα^low^ phenotype. For example, when such pre-cDC were isolated from bone marrow and transferred i.v., both cDC1 and cDC2 progeny were detectable in the spleen of recipient mice (32). Thus, our finding that the reduction in thymic pre-cDC in *Ccr7^−/−^* mice results in a selective deficiency in cDC1, but not cDC2, appears at odds with their capacity to act as common progenitors for cDC. One possible explanation is that as cDC2 can enter the thymus from the periphery as already mature cells, these cells then occupy a finite number of appropriate niches (43). This may then limit the intrathymic generation of cDC2 from colonizing pre-cDC, which may result in their intrathymic skewing toward cDC1 development. Alternatively, pre-cDC that enter the thymus may represent a particular subset of these cells that may already be biased toward cDC1 development. Further comparative analysis of DC progenitors in thymus and peripheral lymphoid tissues may help in discriminating these possibilities. It is also interesting to note that although cDC1 and cDC2 both express CCR7 (21, 44), the cDC defect in *Ccr7^−/−^* mice maps to cDC1 and not cDC2. Thus, redundancy in the chemokine receptors expressed by cDC2 (e.g., CCR2) may still promote their efficient migration to the thymus. In contrast, CCR7 appears to represent a dominant chemokine receptor for intrathymic cDC1 development, although the presence of at least some pre-cDC/cDC1 in the thymus of *Ccr7^−/−^* mice may also suggest compensatory roles for other chemokine receptors, albeit less effectively than CCR7.

In addition, by analyzing the chemokine ligand requirements of thymic DC, we show that CCL21Ser is both essential and sufficient for CCR7-mediated control of thymic cDC1 and their progenitors. Interestingly, that thymic DC require CCL21Ser but not CCL19 may be similar to the requirements of DC in lymph nodes, where DC homeostasis and function were reported to be unaffected in *Ccl19^−/−^* mice (56). It is also worthy to note that in the thymus, CCL21Ser expression has recently been shown to map specifically to mTEC (38), which also control the intrathymic positioning of cDC1 via their expression of XCL1 (57). Taken together, such findings emphasize the importance of mTEC in the regulation of thymic DC and highlight roles for multiple chemokines in both the recruitment (CCL21Ser) and intrathymic positioning (XCL1) processes that take place during thymic cDC1 development. Interestingly, however, although mTEC expression of CCL21 is controlled by LTβR signaling (50), absence of LTβR expression by TEC does not perturb thymic DC numbers (58). Thus, additional receptors expressed by the thymic epithelium may also trigger CCL21 expression to regulate intrathymic cDC. Finally, the paucity of thymic cDC1 in *Ccr7^−/−^* and *Ccl21a^−/−^* mice described in this article may also be important in explaining the importance of CCR7 and its ligands in central tolerance. Indeed, CCL21Ser, but not CCL19, has recently been shown to be important for T cell tolerance in the thymus, where it controls medulla entry of positively selected thymocytes (38). Taken together, these findings indicate that CCR7–CCL21Ser interactions may be important for central tolerance in two separate ways: regulation of thymocyte cortex-to-medulla migration and the regulation of thymic cDC1 availability. In conclusion, our study shows that CCR7 determines cDC1 development in the thymus via a mechanism involving its ligand CCL21Ser and the recruitment of CCR7-expressing pre-cDC. These findings highlight the importance of multiple chemokine receptors in controlling the makeup of the intrathymic DC pool and demonstrate further the key influence of CCR7 on thymus function.
